# Parental Report of Signs of Anxiety and Depression in Children and Adolescents with and Without Disability in Middle- and Low-Income Countries: Meta-analysis of 44 Nationally Representative Cross-Sectional Surveys

**DOI:** 10.1007/s10578-023-01608-8

**Published:** 2023-10-04

**Authors:** Eric Emerson, Gwynnyth Llewellyn

**Affiliations:** 1 Centre for Disability Research and Policy, Faculty of Medicine and Health, University of Sydney, Sydney, NSW 2006 Australia; 2 Centre for Disability Research, Faculty of Health & Medicine, Lancaster University, Lancaster, LA1 4YW UK; 3 Centre of Research Excellence in Disability and Health, University of Melbourne, Melbourne, VIC 3010 Australia

**Keywords:** Disability, Children, Adolescence, Anxiety, Depression, Low and middle-income countries

## Abstract

Population-based studies undertaken in high-income countries have indicated that children and adolescents with disabilities are more likely than their non-disabled peers to experience emotional difficulties such as anxiety and depression. Very little is known about the association between disability and emotional difficulties among children growing up in low and middle-income countries (LMICs). We aimed to estimate the strength of association between disability and two forms of emotional difficulties (anxiety, depression) in a range of LMICs and to determine whether the strength of this relationship was moderated by child age and gender. Secondary analysis of data collected in Round 6 of UNICEF’s Multiple Indicator Cluster Surveys undertaken in 44 LMICs (combined n = 349,421). Data were aggregated across countries by both mixed effects multi-level modelling and restricted maximum likelihood meta-analysis. Young people with disabilities, when compared with their non-disabled peers, were approximately two and a half times more likely to be reported by parents to show daily signs of either anxiety or depression. The level of risk among young people with disabilities was highest in upper middle-income countries and lowest in low-income countries. We estimated that approximately 20% of young people with frequent anxiety or depression also had a disability. All approaches to mental health interventions (from primary prevention to clinical interventions) need to make reasonable accommodations to their services to ensure that the young people with emotional difficulties who also have a disability are not ‘left behind’.

**Supplementary Information:**

The online version contains supplementary material available at 10.1007/s10578-023-01608-8.

## Introduction

A growing number of population-based studies undertaken in high-income countries have indicated that children and adolescents with disabilities are more likely than their non-disabled peers to experience emotional difficulties such as anxiety and depression. [1–5] There is also some evidence to suggest that the association between disability and emotional difficulties may be moderated by gender with particularly high rates of emotional difficulties seen in girls with disabilities during adolescence. [6] Similar results have been reported for gender moderation of the association between disability and low school satisfaction [7, 8] and disability and exposure to cyber victimisation, [9] a possible social determinant of emotional difficulties.

In contrast, few robust epidemiological studies have addressed the mental health of children and young people in low- and middle-income countries (LMICs) [10, 11] and very little is known about the association between disability and emotional difficulties among children growing up in LMICs. For example, a recent systematic review of mental health outcomes among adolescents with chronic illness often associated with disability identified 129 relevant papers. Of these, only five were considered to be at a low risk of methodological bias, all of which were undertaken in high income countries. Of the remaining 124 studies, only 15 compared the emotional well-being of children/adolescents with and without chronic illness in middle-income countries. None were undertaken in low-income countries. Of these 15 studies, 14 used clinic-based samples and 10 had samples sizes of less than 100 children. The majority of the 15 studies reported significantly higher rates of emotional difficulties among children with chronic illness including among children with: asthma in Brazil; [12] type 1 diabetes in China; [13] anaemia or thalassemia in Egypt; [14, 15] asthma in India; [16] sickle cell anaemia or juvenile diabetes mellitus in Nigeria; [17, 18] juvenile rheumatoid arthritis in North Macedonia; [19] diabetes, asthma or epilepsy in Russia; [20] Type 1 diabetes, familial Mediterranean fever or thalassemia in Turkey. [21–23] Missing from this evidence base are any population-based studies of the prevalence of emotional difficulties among children with/without disabilities in LMICs.

It is unsurprising, therefore, that UNICEF in their State of the World’s Children 2021 report (which focused specifically on the mental health of children and adolescents) failed to provide any empirical estimates of the numbers or percentages of mental health problems of children with/without disability. They did, however, draw attention to the importance of collecting data on mental health need in LMICs that were capable of disaggregation by ‘specific groups of children and adolescents … in order to highlight particular vulnerabilities. Most prominently, these included children and adolescents who are detained or incarcerated; refugees and the internally displaced; *those living with disabilities*; and children and adolescents experiencing homelessness’ [p. 147, emphasis added]. [24].

Very recently, de Castro and colleagues [25] published analyses of the association between functional difficulties associated with disability and signs of anxiety and depression among 123,975 adolescents aged 10–17 years in 26 low- and middle-income countries using data extracted from Round 6 of UNICEF’s Multiple Indicator Cluster Surveys (MICS). [26] They reported that compared to adolescents without functional difficulties, those with difficulties were three times more likely to show signs of depression and anxiety with the highest rates evident among adolescents with difficulties in self-care, communicating or accepting changes.

### Aims

1. To estimate the strength of association between disability and signs of two forms of emotional difficulties (anxiety, depression) in a wider range of LMICs.
2. To determine whether the strength of this relationship is moderated by child age and gender.

### Method

We undertook secondary analysis of nationally representative data collected in Round 6 (2017-) of UNICEF’s MICS. [26] Following approval by UNICEF, confidentialised MICS data were downloaded from http://mics.unicef.org/. MICS contains several questionnaire modules. Data used in the present paper were extracted from the household module and the module applied to a randomly selected child age 5–17 living in the household [27]. All MICS questionnaires are translated into the local languages of each country and then back translated as a validity check. All countries used cluster sampling methods to derive samples representative of the national population of mothers and young children. Specific details of the sampling procedure and ethical review arrangements used in each country are freely available in country-level reports at http://mics.unicef.org/. At the end of the download period (17 July, 2023), nationally representative survey data (containing disability data for children aged 5–17) were available for 44 LMICs (17 upper-middle, 17 lower-middle and 10 low-income countries).

### Disability

In Round 6 of MICS a new module was introduced to identify children aged 5–17 with disabilities. The new module, developed by the Washington Group on Disability Statistics (WGDS: http://www.washingtongroup-disability.com/), is based on informant (typically the child’s mother) report of child difficulties in 14 domains (seeing, hearing, walking, self-care, being understood inside the household, being understood outside the household, learning, remembering, focusing, accepting change, making friends, controlling behaviour, anxiety and depression). Four response options were available for all domains other than for anxiety and depression ( [1] ‘*no difficulty*’, [2] ‘*some difficulty*’, [3] ‘*a lot of difficulty*’, [4] ‘*cannot do at all*’; for controlling behaviour the four options were [1] ‘not at all’, [2] ‘the same or less’, [3] ‘more’, [4] ‘a lot more’).

During the development of the module cognitive interviews were undertaken in the United States, India, Montenegro, Belize, Oman and Jamaica in an attempt to ensure that questions were understood similarly in different languages and cultures. [28] Following initial validation of the module in three LMICs, the WGDS recommended using a cut-off to define disability primarily based on the child having at least ‘*a lot of difficulty*’ in one or more domains. [29] We used this recommended cut-off to define child disability and child disabilities associated with the specific functional limitations listed above. However, given our outcomes of interest were levels of reported anxiety and depression among participants with/without disability, we defined disability having at least ‘*a lot of difficulty*’ in one or more of 12 of the 14 domains; the excluded domains for defining disability being anxiety and depression. As a result, children who would have been defined by the WGDS as having a disability *solely* because of scoring above the recommended cut-off on either the anxiety or depression domains were, for the purpose of the present study, classified as not having a disability. For all disability measures the reference group was children without disabilities. Disability data were missing for less than 1% of children.

### Emotional Difficulties

Our measures of emotional difficulties were derived from the two items relating to the anxiety and depression domains contained in the WGDS module for children aged 5–17.

1. ‘*I would like to know how often *(**name**)* seems very anxious, nervous or worried*?’
2. ‘*I would also like to know how often *(**name**)* seems very sad or depressed?*’

Response options were recoded into separate binary variables for signs of anxiety and depression and for signs of co-occurring (comorbid) anxiety and depression (daily vs. less frequently). These data were available for all children with valid disability data.

### Country Characteristics

Given the commonly reported association between child wellbeing and national wealth in low and middle income countries, [30] we used World Bank 2018 country classification as upper middle income, lower middle income and low income. [31] These classifications are based on per capita Gross National Income adjusted for purchasing power parity (pcGNI; expressed as current US$ rates) using the World Bank’s Atlas Method. We also downloaded 2018 Atlas Method pcGNI from the World Bank website in between May 2020 and July 2023. [32, 33].

### Covariates

We included child age (grouped either into a categorical variable of age 5–7, 8–10, 11–13, 14–17 or as a binary variable age 5–12 vs. 13–17) and gender (male/female) as covariates. No data were missing for these two variables. We also included, in some analyses, measures of relative household wealth and highest level of maternal education.

### Relative Household Wealth

MICS data includes a within-country wealth index for each household. To construct the wealth index, principal components analysis is performed by using information on the ownership of consumer goods, dwelling characteristics, water and sanitation, and other characteristics that are related to the household’s wealth, to generate weights for each item. Each household is assigned a wealth score based on the assets owned by that household weighted by factors scores. The wealth index is assumed to capture underlying long-term wealth through information on the household assets. [34, 35] These data were collected in all countries. Data were missing for 0.1% of children with valid disability data.

### Highest Level of Maternal Education

The highest level of education received by each woman in the household was recorded using country-specific categories. We recoded these data into a three-category measure: (1) no education; (2) primary education; (3) secondary or higher-level education. Data were missing for 0.6% of children with valid disability data.

### Approach to Analysis

In the first stage of analysis, we used simple bivariate descriptive statistics to estimate the prevalence of child disability and of emotional difficulties (with 95% confidence intervals) for each country. In the second stage of analysis, we used mixed effects multilevel modelling to investigate the extent to which child disability was associated with emotional wellbeing. We fitted two models to the data; Model 1 controlled for possible confounding effects associated with child gender and age, Model 2 also controlled for possible confounding effects associated with household wealth and highest level of maternal education. Random effects were specified to allow both the intercept and slope of the association between disability and emotional difficulties to vary across countries. These models were fitted to the complete data set and subsamples stratified by country economic classification group. Our selection of random effects models was driven by their capacity to provide estimates of the association between disability and emotional difficulties across countries while allowing for the nature of the relationship to vary between countries. This was considered crucial given the very high probability that any relationship between disability and emotional difficulties is likely to be influenced by cultural practices and socio-economic conditions that vary widely across LMICs. However, random effects models may be subject to bias if independent variables are correlated with unit effects (although Monte Carlo simulations have suggested that this bias may be negligible except in data sets with very small numbers of observations per unit and extremely high correlation between the independent variable and unit effects). [36] Nevertheless, we included in our analyses an alternative method for aggregating data across countries (restricted maximum likelihood meta-analysis of individual country-level estimates) to check for any potential bias in the mixed effects models.

In the final stage of analysis, we used mixed effects multilevel modelling with a full factorial model including all two- and three-way interactions to investigate the possible moderation of the association between disability and emotional difficulties by child age (adolescent or not) and gender. Any significant interaction effects were investigated by inspection of group marginal means.

All analyses were undertaken using Stata 16.1. Country prevalence estimates used the svy command to take account of the clustering of observations within sampling strata and primary sampling units. Multilevel modelling was undertaken using the mepoisson command to generate adjusted prevalence rate ratios (adjusted relative risk). UNICEF’s country-specific child-level weights were used to take account of biases in sampling frames and household and individual level non-response. Given the small amount of missing data, complete case analyses were undertaken.

## Results

### Prevalence of Disability, Anxiety and Depression

Information on the 44 surveys (including sample sizes, response rates and the prevalence of child disability and signs of emotional difficulties) is presented in Table 1. The overall prevalence of disability was 9.0% (95% CI 8.8–9.3, inter country range 1.9–20.3). The overall prevalence of daily signs of anxiety, depression and comorbid anxiety and depression were 9.0% (95% CI 8.6–9.3, inter country range 0.5–24.4), 5.1% (95% CI 4.9–5.2, inter country range 0.3–14.9) and 3.8% (95% CI 3.7–3.9, inter country range 0.2–12.4) respectively. The prevalence of disability, signs of depression and signs of co-morbid anxiety and depression were all significantly negatively correlated with country wealth (disability non-parametric r = − 0.35, p < 0.05; signs of depression non-parametric r= − 0.37, p < 0.05; signs of co-morbid anxiety and depression non-parametric r = − 0.36, p < 0.05). The prevalence of daily signs of anxiety was also negatively correlated with country wealth but was not statistically significant (anxiety non-parametric r = − 0.21, n.s.). Inter-country range within economic classification groups for the prevalence of disability was 15.0% (Costa Rica) − 2.0% (Turkmenistan) in Upper-Middle Income Countries, 17.1% (Ghana) − 1.9% (Vietnam) in Lower-Middle Income Countries and 20.3% (Central African Republic) − 2.4% (Guinea Bissau) in Lower-Middle Income Countries. Corresponding data for signs of anxiety are 16.3% (Iraq) − 0.5% (Turkmenistan), 16.6% (Tunisia) − 0.6% (Vietnam) and 24.4% (Afghanistan) − 2.2% (The Gambia). Corresponding data for signs of depression are 6.6% (Iraq) − 0.4% (Turkmenistan), 13.1% (Samoa) − 0.3% (Vietnam) and 14.9% (Afghanistan) − 1.6% (The Gambia).

**Table 1:** Survey details and prevalence of child disabilities and emotional wellbeing by Country

	Year of survey	pcGNI (2018)	HDI (2018)	Response rate (%)	Sample size	Prevalence (with 95% CI)			
						Disability	Anxiety	Depression	Comorbid Anxiety and Depression
*Upper Middle-Income*									
Argentina	2019/20	$12,370	0.842	81.8	6,378	6.8% (6.0−8.1)	8.2% (7.0−9.7)	3.0% (2.2–4.2)	2.1% (1.4–3.1)
Costa Rica	2018	$11,590	0.794	87.1	3,952	15.0% (13.3–16.9)	8.5% (7.4–9.9)	2.2% (1.6–3.1)	1.3% (0.8− 2.0)
Montenegro	2018/19	$8,430	0.816	60.5	1,610	3.2% (1.8–5.8)	10.6% (5.3–20.1)	3.8% (2.2–6.5)	2.5% (1.6–3.8)
Dominican Republic	2019	$7,760	0.751	99.0	13,114	6.2% (5.6–6.9)	6.1% (5.4−7.0)	2.2% (1.8–2.7)	1.5% (1.2–1.9)
Cuba	2019	$7,480	0.781	98.5	4,293	4.1% (3.0− 5.6)	8.1% (6.8–9.7)	4.5% (3.5–5.8)	4.0% (3.1–5.2)
Turkmenistan	2019	$6,740	0.710	97.1	3,726	2.0% (1.6–2.6)	0.5% (0.3–0.8)	0.4% (0.2–0.8)	0.2% (0.1–0.4)
Serbia	2019	$6,400	0.799	80.8	2,706	5.5% (4.3–7.1)	4.1% (3.1–5.4)	2.5% (1.8–3.5)	1.9% (1.3–2.7)
Guyana	2019/20	$6,290	0.680	89.3	3,192	10.4% (8.4–12.8)	9.5% (7.9–11.4)	3.9% (3.1−5.0)	2.3% (1.7-3.0)
Fiji	2021	$5,910	0.724	96.8	2,861	8.4% (7.0− 10.1)	2.9% (2.2–3.8)	1.5% (1.0-2.3)	1.0% (0.6–1.7)
Belarus	2019	$5,700	0.823	95.6	2,731	4.0% (3.0−5.2)	1.1% (0.6–1.9)	0.5% (0.3−1.0)	0.3% (0.1–0.8)
North Macedonia	2018/19	$5,470	0.759	86.8	1,427	2.6% (1.6–4.2)	9.3% (6.8–12.6)	1.8% (0.8–3.8)	1.6% (0.7–3.6)
Tuvalu	2019/20	$5,430	n/a	96.8	434	11.8% (8.7–15.8)	1.0% (0.4–2.4)	0.8% (0.3–2.2)	0.4% (0.1–1.4)
Suriname	2018	$5,210	0.724	82.7	3,850	10.8% (9.7–11.5)	4.4% (3.9–5.1)	2.0% (1.6–2.5)	1.4% (1.0−1.9)
Iraq	2018	$5,040	0.689	99.4	15,542	7.1% (6.3–8.1)	16.3% (15.1–17.6)	6.6% (5.9–7.5)	4.8% (4.2–5.5)
Georgia	2018	$4,450	0.786	83.4	3,715	5.8% (4.6–7.3)	4.4% (3.5–5.6)	1.6% (1.2–2.3)	1.1% (0.7–1.7)
Kosovo	2019/20	$4,340	0.739	80.1	2,355	3.7% (2.8–4.9)	5.5% (4.4–6.7)	2.1% (1.5− 3.0)	1.2% (0.8–1.9)
Tonga	2019	$4,300	0.717	96.1	1,626	5.1% (3.7–6.9)	4.3% (3.2–5.9)	4.0% (2.5–6.3)	1.8% (1.1− 3.0)
*Lower Middle-Income*									
Palestine	2019/20	$4,180	0.708	95.7	5,317	6.4% (5.5–7.5)	10.2% (9.2–11.5)	2.5% (2.0–3.0)	1.7% (1.4–2.2)
Samoa	2019/20	$4,020	0.704	93.0	2,237	8.2% (6.6–10.0)	14.7% (11.8–18.0)	13.1% (10.4–16.4)	11.4% (8.8–14.7)
Algeria	2018	$3,980	0.746	94.7	16,353	9.4% (8.5–10.3)	13.2% (12.3–14.2)	4.5% (4.0–5.0)	3.7% (3.3–4.2)
Mongolia	2018	$3,660	0.735	95.7	7,397	5.7% (4.9–6.6)	2.2% (1.7–2.9)	1.1% (0.7–1.6)	0.6% (0.4–0.9)
Tunisia	2018	$3,500	0.739	96.9	4,889	12.8% (11.9–13.7)	16.6% (15.6–17.7)	4.4% (3.9-5.0)	3.6% (3.1–4.2)
Kiribati	2018/19	$3,140	0.623	98.2	2,248	12.6% (10.7–14.6)	10.0% (8.1–12.3)	4.6% (3.5–5.9)	1.3% (0.8–2.1)
Vietnam	2020/21	$2,380	0.693	97.3	7,003	1.9% (1.6–2.4)	0.6% (0.4–0.8)	0.3% (0.2–0.5)	0.2% (0.1–0.4)
Honduras	2019	$2,320	0.626	90.5	11,863	12.5% (11.7–13.3)	4.7% (4.2–5.3)	2.8% (2.4–3.2)	1.5% (1.3–1.9)
Ghana	2017/18	$2,130	0.596	99.2	8,932	17.1% (15.5–18.7)	4.2% (3.4–5.2)	2.9% (2.3–3.7)	2.2% (1.7–2.9)
Uzbekistan	2021/22	$2,120	0.720	99.6	9,417	6.5% (5.4–7.9)	12.9% (11.0−15.2)	3.5% (2.6–4.6)	2.2% (1.5–3.2)
Nigeria	2021	$1,980	0.531	97.7	22,706	7.3% (6.6–8.1)	8.1% (7.3–8.8)	6.1% (5.5–6.8)	4.7% (4.1–5.3
Sao Tome & Principe	2019	$1,870	0.624	96.9	2,171	13.4% (11.4–15.5)	9.1% (7.7–10.8)	3.5% (2.6–4.6)	2.1% (1.5–2.9)
Zimbabwe	2018/19	$1,790	0.563	96.3	7,021	7.7% (6.9–8.6)	2.6% (2.1–3.1)	1.9% (1.5–2.3)	1.0% (0.8–1.4)
Bangladesh	2019	$1,750	0.614	96.4	39,474	5.5% (5.2–5.9)	3.2% (2.9–3.5)	3.7% (3.5-4.0)	2.6% (2.4–2.8)
Lesotho	2018	$1,390	0.518	90.1	4,971	7.4% (6.5–8.4)	1.3% (0.9–1.8)	0.6% (0.4–0.9)	0.4% (0.3–0.7)
Kyrgyz Republic	2018	$1,220	0.674	98.4	3,890	3.6% (3.1–4.3)	5.3% (4.6-6.0)	1.6% (1.2−2.0)	0.7% (0.3–1.1)
Nepal	2019	$970	0.579	99.3	7,775	5.2% (4.5−6.0)	10.6% (9.6–11.6)	2.4% (1.9−3.0)	2.0% (1.6–2.5)
*Low-Income*									
Guinea-Bissau	2018/19	$750	0.461	99.6	5,836	2.4% (1.8–3.1)	14.1% (12.3–16.2)	4.8% (3.9–5.8)	4.2% (3.4–5.2)
The Gambia	2018	$710	0.466	95.9	5,685	7.8% (6.6–9.1)	2.2% (1.7–2.8)	1.6% (1.2–2.1)	0.8% (0.5–1.3)
Chad	2019	$680	0.397	99.4	14,779	9.5% (8.6–10.4)	19.7% (18.5–21.0)	13.2% (12.2–14.3)	10.3% (9.4–11.4)
Togo	2017	$660	0.513	96.3	4,925	14.8% (13.2–16.5)	8.4% (7.3–9.7)	5.0% (4.1–6.2)	4.0% (3.2–5.1)
Afghanistan	2022/23	$520	0.483	98.8	20,222	15.8% (14.8–17.0)	24.4% (23.0-25.9)	14.9% (13.8–16.0)	12.4% (11.4–13.4)
Madagascar	2018	$510	0.521	94.1	11,936	11.1% (10.2–12.1)	3.7% (3.2–4.2)	3.1% (2.7–3.6)	1.7% (1.4−2.0)
DR Congo	2017/18	$490	0.459	99.8	13,997	9.2% (8.0−10.6)	11.6% (10.2–13.2)	6.7% (5.8–7.8)	5.5% (4.5–6.5)
Sierra Leone	2017	$490	0.438	99.5	10,958	10.6% (10.0−11.1)	12.7% (12.1–13.3)	9.2% (8.7–9.7)	6.5% (6.0–7.0)
Central African Republic	2018/19	$490	0.381	96.3	5,941	20.3% (18.6–22.1)	15.4% (14.0–17.0)	9.5% (8.4–10.9)	7.0% (5.9–8.2)
Malawi	2019/20	$350	0.485	97.9	17,707	10.5% (9.8–11.2)	4.6% (4.1–5.1)	3.4% (3.0−3.9)	2.7% (2.4–3.1)

Sample sizes are unweighted and only include children for who valid data on disability status are available

### Association Between Disability and Emotional Difficulties

**Fig. 1:**
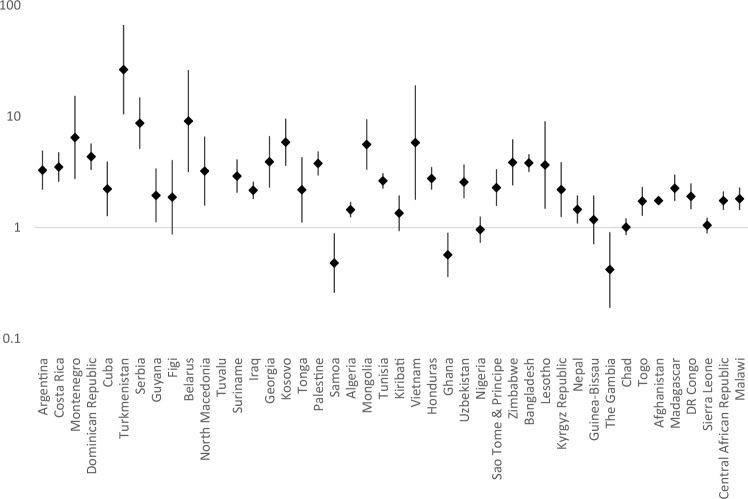
Prevalence rate ratios (adjusted for age and gender, plotted on logarithmic scale) with 95% Confidence Intervals for Risk of Anxiety Among Children with Disabilities

**Fig. 2:**
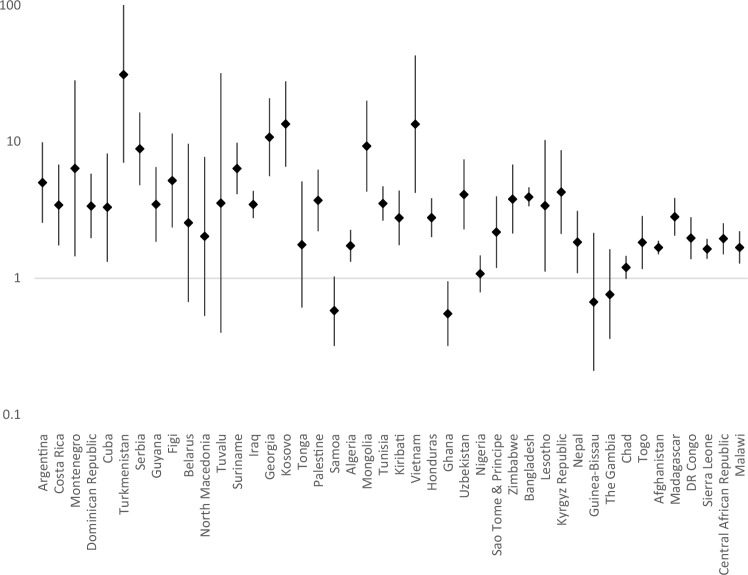
Prevalence rate ratios (adjusted for age and gender, plotted on logarithmic scale) with 95% Confidence Intervals for Risk of Depression Among Children with Disabilities

**Fig. 3:**
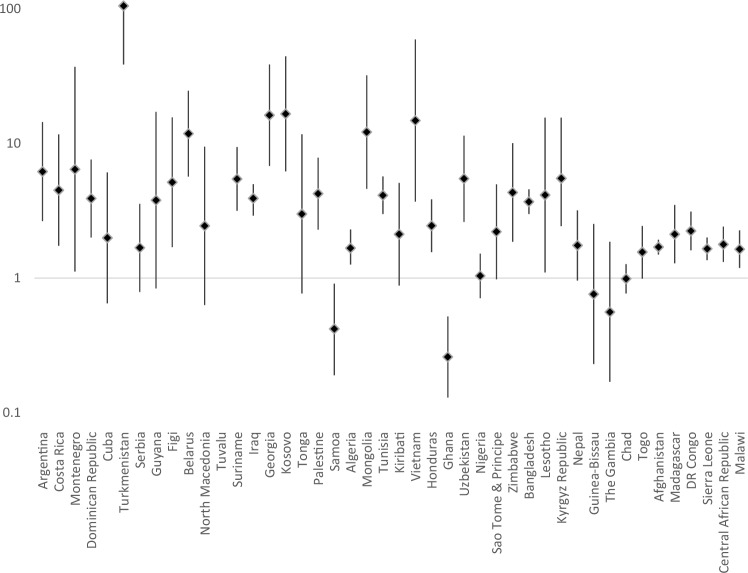
Prevalence rate ratios (adjusted for age and gender, plotted on logarithmic scale) with 95% Confidence Intervals for Risk of Comorbid Anxiety and Depression Among Children with Disabilities

Country-level data on the prevalence of signs of emotional difficulties among children and young people with/without disabilities are presented in Supplementary Table 1 (for signs of anxiety and depression) and Supplementary Table 2 (for signs of comorbid anxiety and depression). Prevalence rate ratios for each country are presented in Fig. 1, 2 and 3. For signs of anxiety, prevalence was higher among children with disabilities in 39 of the 44 countries (significantly so in 34 countries), it was significantly lower in three countries (Samoa, Ghana, The Gambia). For signs of depression, prevalence was higher among children with disabilities in 39 of the 44 countries (significantly so in 34 countries), it was significantly lower in one country (Ghana). For signs of co-morbid anxiety and depression, prevalence was higher among children with disabilities in 38 of the 44 countries (significantly so in 28 countries), it was significantly lower in two countries (Ghana and Samoa).

Mixed effects multilevel modelling (controlling for child age and gender) estimated that children with disabilities, when compared with their non-disabled peers, were over twice as likely to shown daily signs of anxiety (APRR = 2.26, 95% CI 1.85–2.77, p < 0.001), were over two and a half times more likely to show daily signs of depression (APRR = 2.74, 95% CI 2.18–3.46, p < 0.001) and were over three times more likely to show daily signs of comorbid anxiety and depression (APRR = 3.20, 95% CI 2.45–4.17, p < 0.001). Additionally controlling for between-group differences in relative household wealth and highest level of maternal education had little effect on these estimates (anxiety APRR = 2.25, 95% CI 1.84–2.75, p < 0.001; depression APRR = 2.73, 95% CI 2.17–3.44, p < 0.001; co-morbid anxiety and depression APRR = 2.72, 95% CI 2.04–3.63, p < 0.001).

Aggregating data across countries using meta-analysis (controlling for age and gender) gave slightly larger effect sizes for signs of depression (APRR = 2.83, 95% CI 2.25–3.56, I^2^ = 96.0%, p < 0.001). For signs of anxiety and signs of comorbid anxiety and depression it was not possible to estimate standard errors for one country (Tuvalu) due to a zero cell in the basic 2 × 2 categorisation of disability by anxiety. Dropping Tuvalu from the analyses again resulted in slightly larger effect sizes for meta-analysis when compared with estimates derived from mixed effects multilevel modelling excluding Tuvalu for signs of anxiety (meta-analysis APRR = 2.36, 95% CI 1.92–2.92, I^2^ = 93.6%, p < 0.001); multilevel APRR = 2.28, 95% CI 1.87–2.79, p < 0.001) and slightly lower for signs of comorbid anxiety and depression (meta-analysis APRR = 2.97, 95% CI 2.18–4.06, I^2^ = 95.0%, p < 0.001); multilevel APRR = 3.21, 95% CI 2.45–4.20, p < 0.001).

Stratifying mixed effects multilevel modelling analyses by country economic classification group indicated that effect sizes for adjusted relative risk of signs of anxiety, depression and comorbid anxiety and depression among young people with disabilities increased with each increase in country wealth classification (Table 2). In addition, we examined the non-parametric correlation between country level effect sizes and pcGNI. These analyses also demonstrated robust associations between country pcGNI and country level effect sizes for adjusted relative risk of signs of anxiety (r = 0.51, p < 0.001), signs of depression (r = 0.48, p < 0.001) and signs of comorbid anxiety and depression among young people with disabilities (r = 0.53, p < 0.001). The estimated percentage of children and young people with frequent anxiety who also had other disabilities was 18% (95% CI 17–20%) in upper middle-income countries, 15% (95% CI 13−17%) in lower middle-income countries and 19% (95% CI 17−21%) in low-income countries. The corresponding percentages for depression were 22% (95% CI 19−25%), 16% (95% CI 14–18%) and 20% (95% CI 18−22%) and for comorbid anxiety and depression were 24% (95% CI 20−27%), 15% (95% CI 13−17%) and 19% (95% CI 18-21%).

**Table 2:** Prevalence and adjusted prevalence rate ratios for risk of emotional difficulties among children with disabilities

	Prevalence		APRR	
	Disability	No Disability	Model 1	Model 2
Anxiety				
Upper-middle income	21.1% (18.6–23.9)	6.9% (6.5–7.3)	3.40*** (2.60–4.44)	3.34*** (2.56–4.37)
Lower-middle income	12.2% (10.1–14.7)	5.8% (5.4–6.4)	2.07*** (1.48–2.88)	2.07*** (1.48–2.90)
Low income	20.6% (18.8–22.4)	12.0% (11.5–12.4)	1.45** (1.14–1.85)	1.45** (1.14–1.85)
Depression				
Upper-middle income	10.8% (9.3–12.5)	2.7% (2.5-3.0)	4.55*** (3.40–6.08)	4.45*** (3.38–5.87)
Lower-middle income	7.3% (6.6–8.1)	3.2% 3.1–3.4	2.57*** (1.77–3.72)	2.55*** (1.75–3.70)
Low income	13.6% (12.6–14.7)	7.4% (7.0–7.9)	1.64*** (1.36–1.97)	1.63*** (1.35–1.97)
Anxiety and depression				
Upper-middle income	8.0% (6.6–9.6)	1.9% (1.7–2.0)	4.77*** (3.17–7.19)	4.67*** (3.14–6.93)
Lower-middle income	4.9% (4.2–5.6)	2.4% (2.2–2.5)	2.58*** (1.59–4.17)	2.57*** (1.58–4.16)
Low income	10.2% (9.3–11.2)	5.8% (5.5–6.1)	1.53*** (1.26–1.87)	1.54*** (1.26–1.87)

### Moderation by Child Age and Gender

**Fig. 4:**
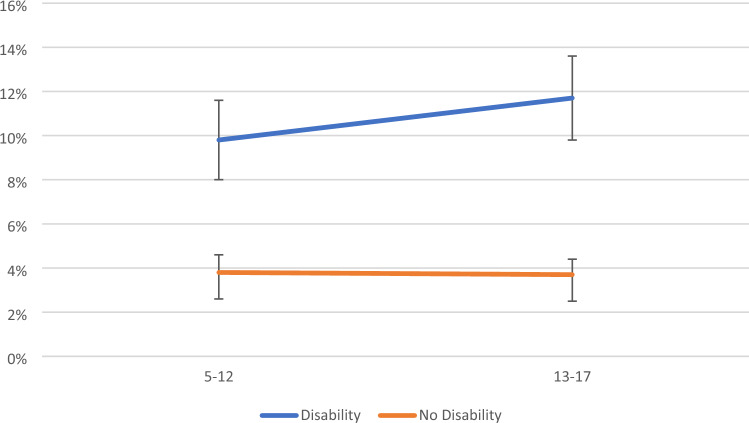
Estimated marginal means for depression (with 95% CIs) by disability status and age

**Fig. 5:**
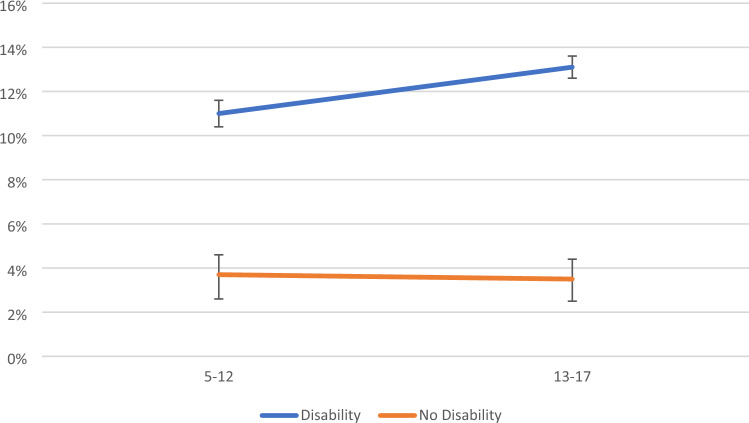
Estimated marginal means for comorbid anxiety and depression (with 95% CIs) by disability status and age

For signs of anxiety, no two- or three-way interaction effects were statistically significant. For signs of depression, there was a significant two-way interaction effects between disability and age (APRR = 1.31 (95% CI 1.12–1.54), p < 0.001). Inspection of marginal means indicated that the difference between young people with/without disabilities increased from 6.0% points in childhood to 8.0% points in adolescence (Fig. 4). For signs of comorbid anxiety and depression, there was also a significant two-way interaction effects between disability and age (APRR = 1.25 (95% CI 1.06–1.48), p < 0.01). Inspection of marginal means indicated that the difference between young people with/without disabilities increased from 7.3% points in childhood to 9.6% points in adolescence (Fig. 5).

## Discussion

Our analysis of the emotional wellbeing on 349,421 children and young people aged 5–17 across 44 LMICs indicated that: (1) children and young people with disabilities, when compared with their non-disabled peers, were over twice as likely to show daily signs of anxiety, over two and a half times more likely to show daily signs of depression and over three times more likely to show signs of comorbid anxiety and depression; (2) the adjusted relative risk of both signs of anxiety and signs of depression among participants with disabilities was highest in upper middle-income countries and lowest in low-income countries; (3) there was a significant age by disability interaction indicating significantly greater disability-based inequalities in signs of depression and signs of comorbid anxiety and depression during adolescence when compared to earlier childhood; and (4) approximately one in five children and young people age 5–17 years with frequent signs of emotional difficulties in LMICs also had another disability.

Our results represent a significant contribution to knowledge in two ways. First, the use of recently collected nationally representative data with high response rates and using a recently developed validated measure of child disability from 44 LMICs constitutes the most robust investigation of the association between child disability and emotional difficulties in LMICs undertaken to date. Specifically, it builds on the excellent work of de Castro and colleagues [25] by including a wider range of LMICS (44 opposed to 26 countries), data on younger children, an investigation of the possible moderating effects of age and gender and the use of arguably more robust statistical methods (mixed effects multilevel modelling and restricted maximum likelihood meta-analysis). Second, our finding of a systematic association between country economic classification (and country pcGNI) and effect sizes for adjusted relative risk of both signs of anxiety, depression and comorbid anxiety and depression among young people with disabilities is, to our knowledge, novel.

The clear implication of these results is the need for all approaches to mental health interventions in LMICs (from primary prevention to clinical interventions) to make reasonable accommodations to ensure that the 20% of young people with emotional difficulties who also have other disabilities are not ‘left behind’. Such an approach would be fully consistent with the application of proportionate universalism in addressing health inequities. [37].

Our finding that the strength of relationship between disability and signs of emotional difficulties is robustly related to country wealth (the relationship being stronger in wealthier LMICs) warrants further research on the possible impact of country wealth on two broad pathways linking disability and wellbeing. The first would focus on the investigation between country wealth and differential rates of exposure of young people with disabilities to established social determinants of poorer mental health (e.g., discrimination, violence, social exclusion). [38] The second would focus on the investigation between country wealth and differential access of young people with disabilities to resources associated with greater resilience if exposed to adversities (e.g., social support, effective and timely health care). We are not aware of any existing evidence that addresses either issue. The finding that the highest prevalence of anxiety and depression in Upper–Middle countries was in Iraq and in Low Income countries was in Afghanistan that are both countries with significant recent conflict and natural disasters requires further investigation, noting that adults with disabilities are disproportionally more severely effected in humanitarian emergencies, this may also be the case for children and young people with disabilities. [39].

The results of our study need to be considered in light of four main limitations. First, the identification of child disability in national health and social surveys is a complex process that runs the risk of under-identification of child disability in poorer households/communities. [40] Recent research has suggested that this may be the case with the new WGDS child disability module implemented in MICS in relation to functional limitations in learning. [41] Second, the data used are cross-sectional and, as such, cannot be used to determine causal pathways between child disability and signs of emotional wellbeing. Third, our measures of anxiety and depression are based on caretaker (primarily maternal) report of the frequency of the child or young person showing signs of anxiety and depression. The association between these measures and diagnosable episodes of anxiety and depression are unknown. Finally, while cognitive interviews were used during the development of the WGDS module on child functioning to assess cross cultural validity, further research is required to ensure validity in the very wide range of countries and cultures implementing MICS6.

## Summary

Evidence generated in high-income countries has indicated that children and adolescents with disabilities are more likely than their non-disabled peers to experience emotional difficulties. However, until very recently very little was known about the association between disability and emotional difficulties among children growing up in LMICs. In 2023 de Castro and colleagues [25] published analyses of the association between functional difficulties associated with disability and signs of anxiety and depression among adolescents aged 10–17 years in 26 LMICs using data extracted from Round 6 of UNICEF’s MICS. They reported that compared to adolescents without functional difficulties, those with difficulties were three times more likely to show signs of depression and anxiety with the highest rates evident among adolescents with difficulties in self-care, communicating or accepting changes.

In order to add to this evidence base we undertook secondary analysis of data collected in Round 6 of UNICEF’s Multiple Indicator Cluster Surveys undertaken in 44 LMICs (combined n=349,421). Our aims were: (1) to estimate the strength of association between disability and two forms of emotional difficulties (anxiety, depression); and (2) to determine whether the strength of this relationship was moderated by child age and gender. Data were aggregated across countries by both mixed effects multi-level modelling and restricted maximum likelihood meta-analysis.

Our results indicated that young people with disabilities, when compared with their non-disabled peers, were approximately two and a half times more likely to be reported by parents to show daily signs of either anxiety or depression. The level of risk among young people with disabilities was highest in upper middle-income countries and lowest in low-income countries. There was no evidence that the association between disability and emotional difficulties was moderated by gender. However, for signs of depression and comorbid anxiety and depression, there was evidence that the association between disability and emotional difficulties was stronger among adolescents than younger children Overall, we estimated that approximately 20% of young people with frequent anxiety or depression also had a disability.

As a result, it is important that all approaches to mental health interventions (from primary prevention to clinical interventions) need to make reasonable accommodations to their services to ensure that the young people with emotional difficulties who also have a disability are not ‘left behind’ but have an equal opportunity to access and benefit from.

## Supplementary Information

Below is the link to the electronic supplementary material.

Supplementary material 1 (DOCX 38.3 kb)

## Data Availability

Following approval by UNICEF the data are freely available at https://mics.unicef.org/surveys.
