# Supplementary material for: Parental Report of Signs of Anxiety and Depression in Children and Adolescents with and Without Disability in Middle- and Low-Income Countries: Meta-analysis of 44 Nationally Representative Cross-Sectional Surveys
Source: Child Psychiatry Hum Dev. 2023 Oct 4;56(4):895–906. doi: 10.1007/s10578-023-01608-8 (PMC12289834; doi:10.1007/s10578-023-01608-8)
Supplement: Supplementary file 1 — Supplementary material 1 (DOCX 38.3 kb) [file 10578_2023_1608_MOESM1_ESM.docx]

Supplementary Materials

| Supplementary Table 1: Prevalence of Anxiety and Depression Among Young People with Disabilities | | | | | | | | |
| --- | --- | --- | --- | --- | --- | --- | --- | --- |
|  | Anxiety | | | | Depression | | | |
|  | Prevalence (with 95% CI) | | APRR (with 95% CI) | | Prevalence (with 95% CI) | | APRR (with 95% CI) | |
|  | Disability | No Disability | APRR1 | APRR2 | Disability | No Disability | APRR1 | APRR2 |
| Argentina | 23.4%  (16.4-32.2) | 7.1%  (5.9-8.6) | 3.29***  (2.20-4.94) | 3.23***  (2.17-4.81) | 11.8%  (6.1-21.6) | 2.4%  (1.6-3.4) | 5.02***  (2.55-9.91) | 4.56***  (2.43-8.55) |
| Costa Rica | 21.7%  (17.3-26.8) | 6.1%  (5.0-7.5) | 3.51***  (2.59-4.76) | 3.44***  (2.50-4.73) | 5.7%  (3.4-9.3) | 1.6%  (1.1-2.5) | 3.44***  (1.74-6.80) | 3.30**  (1.59-6.84) |
| Montenegro | 37.6%  (24.9-52.2) | 9.6%  (4.7-18.6) | 6.47***  (2.74-15.32) | 4.93***  (2.47-9.87) | 15.1%  (8.0-26.8) | 3.3%  (1.9-5.6) | 6.38*  (1.45-28.1) | 5.37*  (1.46-22.52) |
| Dominican Republic | 22.1%  (17.3-27.8) | 5.0%  (4.3-5.9) | 4.35***  (3.31-5.71) | 4.31***  (3.28-5.66) | 6.4%  (3.9-10.6) | 1.9%  (1.6-2.3) | 3.38***  (1.97-5.82) | 3.39***  (1.95-5.90) |
| Cuba | 18.4%  (10.2-30.8) | 7.7%  (6.4-9.2) | 2.23**  (1.27-3.93) | 2.13*  (1.19-3.82) | 13.6%  (5.1-31.9) | 4.1%  (3.2-5.4) | 3.31*  (1.34-8.20) | 3.01*  (1.20-7.54) |
| Turkmenistan | 8.4%  (4.3-15.8) | 0.4%  (0.2-0.6) | 26.41***  (10.46-66.69) | 21.93***  (8.25-58.33) | 7.5%  (3.6-14.9) | 0.3%  (0.1-0.6) | 31.00***  (7.02-136.85) | 38.14***  (13.92-104.47) |
| Serbia | 23.8%  (15.6-34.6) | 2.9%  (2.1-4.1) | 8.71***  (5.11-14.86) | 5.91***  (3.18-10.96) | 15.0%  (8.8-24.5) | 1.8%  (1.2-2.7) | 8.87***  (4.80-16.37) | 5.57***  (2.78-11.15) |
| Guyana | 16.8%  (9.9-27.1) | 8.7%  (7.2-10.4) | 1.95*  (1.12-3.41) | 1.79  (0.97-3.30) | 10.8%  (5.8-19.0) | 3.1%  (2.4-4.1) | 3.48***  (1.85-6.52) | 3.61***  (2.05-6.36) |
| Fiji | 5.0%  (2.6-9.5) | 2.6%  (1.9-3.6) | 1.88  (0.87-4.05) | 2.01  (0.95-4.23 | 5.8%  (3.1-10.5) | 1.1%  (0.7-1.9) | 5.19***  (2.35-11.46) | 5.88***  (2.85-12.14) |
| Belarus | 6.8%  (2.5-17.4) | 0.9%  (0.5-1.6) | 9.10***  (3.16-26.11) | 9.87***  (3.49-27.95) | 1.2%  (0.4-3.1) | 0.5%  (0.2-1.0) | 2.55  (0.67-9.66) | 2.67  (0.70-10.21) |
| North Macedonia | 30.7%  (12.3-58.2) | 8.7%  (6.3-12.1) | 3.23**  (1.58-6.60) | 3.37**  (1.42-7.96) | 2.8%  (0.8-9.3) | 1.7%  (0.8-3.9) | 2.03  (0.53-7.74) | 1.87  (0.42-8.27) |
| Tuvalu | 0.0%  (0.0-7.1**)** | 1.1%  (0.4-2.8) | n/a | n/a | 2.0%  (0.3-13.1) | 0.7%  (0.2-2.2) | 3.55  (0.40-31.8) | 2.55  (0.10-62.58) |
| Suriname | 8.9%  (6.5-11.9) | 3.6%  (3.1-4.4) | 2.91***  (2.07-4.10) | 2.84***  (2.01-4.03) | 6.5%  (4.7-9.5) | 1.5%  (1.1-2.0) | 6.37***  (4.14-9.82) | 6.28***  (4.01-9.83) |
| Iraq | 33.9%  (20.0-39.2) | 15.0%  (13.8-16.3) | 2.17***  (1.81-2.59) | 2.17***  (1.83-2.57) | 19.4%  (15.7-23.7) | 5.6%  (4.9-6.5) | 3.47***  (2.76-4.37) | 3.36***  (2.67-4.23) |
| Georgia | 15.0%  (8.7-24.6) | 3.8%  (3.0-4.9) | 3.91***  (2.29-6.66) | 3.96***  (2.33-6.71) | 10.9%  (5.4-20.6) | 1.1%  (0.7-1.5) | 10.79***  (5.59-20.82) | 10.69***  (5.20-21.96) |
| Kosovo | 27.1%  (17.6-39.3) | 4.6%  (3.6-5.8) | 5.87***  (3.61-9.53) | 5.91***  (3.62-9.66) | 19.8%  (11.5-31.9) | 1.4%  (0.9-2.2) | 13.48***  (6.57-27.66) | 12.65***  (6.55-24.43) |
| Tonga | 9.0%  (4.6-16.8) | 4.1%  (3.0-5.6) | 2.19*  (1.11-4.31) | 2.16*  (1.10-4.24) | 7.0%  (3.0-15.6) | 3.8%  (2.3-6.2) | 1.76  (0.61-5.12) | 1.65  (0.64-4.29) |
| Palestine | 34.4%  (27.3-42.3) | 8.6%  (7.6-9.7) | 3.78***  (2.95-4.84) | 3.83***  (2.98-4.93) | 7.8%  (4.9-12.3) | 2.1%  (1.7-2.6) | 3.72***  (2.21-6.25) | 3.58***  (2.10-6.12) |
| Samoa | 7.3%  (4.2-12.5) | 15.3%  (12.2-19.0) | 0.48*  (0.26-0.89) | 0.49*  (0.26-0.91) | 7.8%  (4.6-13.1) | 13.6%  (10.7-17.2) | 0.58  (0.32-1.03) | 2.75***  (1.98-3.83) |
| Algeria | 18.6%  (16.0-21.6) | 12.6%  (11.7-13.6) | 1.45***  (1.24-1.710 | 1.45***  (1.23-1.70) | 7.3%  (5.7-9.4) | 4.2%  (3.7-4.7) | 1.73***  (1.32-2.26) | 1.70***  (1.30-2.22) |
| Mongolia | 9.6%  (5.8-15.4) | 1.7%  (1.3-2.3) | 5.60***  (3.32-9.46) | 5.64***  (3.35-9.49) | 6.7%  (3.5-12.4) | 0.7%  (0.5-1.1) | 9.28***  (4.31-20.00) | 9.35***  (4.89-17.89) |
| Tunisia | 33.6%  (29.9-37.5) | 14.1%  (13.1-15.2) | 2.64***  (2.24-3.09) | 2.61***  (2.22-3.06) | 11.0%  (8.7-13.7) | 3.5%  (3.0-4.1) | 3.53***  (2.65-4.71) | 3.43***  (2.56-4.59) |
| Kiribati | 13.4%  (9.0-19.6) | 9.4%  (7.6-11.6) | 1.35  (0.93-1.95) | 1.32  (0.91-1.91) | 10.2%  (6.7-15.1) | 3.8%  (2.8-5.2) | 2.77***  (1.75-4.39) | 2.80***  (1.72-4.57) |
| Vietnam | 3.1%  (1.0-8.7) | 0.5%  (0.4-0.8) | 5.82**  (1.78-19.09) | 5.57**  (1.70-18.31) | 3.0%  (0.9-9.3) | 0.2%  (0.1-0.4) | 13.45***  (4.22-42.84) | 12.59***  (4.07-38.91) |
| Honduras | 10.8%  (8.9-13.0) | 3.9%  (3.4-4.4) | 2.77***  (2.20-3.50) | 2.74***  (2.18-3.45) | 6.2%  (4.6-8.3) | 2.3%  (1.9-2.7) | 2.78***  (2.00-3.86) | 2.75***  (1.98-3.82) |
| Ghana | 2.6%  (1.7-3.8) | 4.6%  (3.6-5.8) | 0.57*  (0.36-0.90) | 0.58*  (0.37-0.91) | 1.7%  (1.1-2.8) | 3.2%  (2.5-4.1) | 0.55*  (0.32-0.95) | 0.55*  (0.32-0.93) |
| Uzbekistan | 30.8%  (22.8-40.3) | 11.6%  (9.7-13.8) | 2.57***  (1.84-3.69) | 2.43***  (1.76-3.36) | 12.4%  (7.7-19.5) | 2.9%  (2.1-4.1) | 4.11***  (2.28-7.41) | 3.80***  (2.21-6.52) |
| Nigeria | 7.8%  (6.0-10.0) | 8.1%  (7.3-8.9) | 0.96  (0.73-1.26) | 0.91  (0.69-1.21) | 6.6%  (4.9-8.8) | 6.1%  (5.4-6.8) | 1.08  (0.79-1.47) | 1.04  (0.75-1.42) |
| Sao Tome & Principe | 18.0%  (12.9-24.6) | 7.9%  (6.4-9.7) | 2.29***  (1.57-3.35) | 2.19***  (1.50-3.19) | 6.7%  (4.1-10.6) | 3.0%  (2.1-4.3) | 2.18*  (1.19-3.98) | 1.99*  (1.06-3.74) |
| Zimbabwe | 8.1%  (5.3-12.0) | 2.1%  (1.7-2.6) | 3.87***  (2.40-6.24) | 3.93***  (2.38-6.47) | 5.9%  (3.5-9.7) | 1.5%  (1.2-2.0) | 3.80***  (2.13-6.80) | 3.49***  (1.87-6.51) |
| Bangladesh | 10.4%  (8.8-12.2) | 2.8%  (2.5-3.0) | 3.82***  (3.17-4.58) | 3.90***  (3.24-4.68) | 12.8%  (11.0-14.7) | 3.2%  (3.0-3.4) | 3.94***  (3.37-4.64) | 3.96***  (3.36-4.68) |
| Lesotho | 3.9%  (1.5-9.6) | 1.1%  (0.8-1.5) | 3.66**  (1.48-9.07) | 4.04**  (1.67-9.79) | 1.8%  (0.6-4.8) | 0.5%  (0.3-0.8) | 3.40*  (1.12-10.28) | 3.70*  (1.22-11.23) |
| Kyrgyz Republic | 10.1%  (6.2-16.4) | 5.1%  (4.4-5.8) | 2.20**  (1.25-3.88) | 2.05*  (1.14-3.69) | 7.4%  (4.0-12.9) | 1.4%  (1.1-1.8) | 4.28***  (2.11-8.67) | 3.84***  (1.82-8.07) |
| Nepal | 15.3%  (11.5-20.1) | 10.3%  (9.3-11.3) | 1.46*  (1.09-1.95) | 1.44*  (1.08-1.93) | 4.2%  (2.6-6.7) | 2.3%  (1.8-2.9) | 1.84*  (1.09-3.11) | 1.82*  (1.07-3.09) |
| Guinea-Bissau | 16.7%  (9.7-26.8) | 14.1%  (12.2-16.2) | 1.18  (0.71-1.95) | 1.18  (0.71-1.95) | 3.3%  (1.0-10.0) | 4.8%  (3.9-5.9) | 0.67  (0.21-2.15) | 0.64  (0.19-2.09) |
| The Gambia | 0.9%  (0.5-2.0) | 2.3%  (1.7-3.0) | 0.42*  (0.19-0.91) | 0.43*  (0.20-0.93) | 1.3%  (0.6-2.6) | 1.6%  (1.2-2.1) | 0.76  (0.36-1.63) | 0.75  (0.34-1.64) |
| Chad | 20.1%  (17.0-23.6)) | 19.7%  (18.4-20.9) | 1.01  (0.86-1.21) | 1.02  (0.86-1.20) | 15.6%  (12.9-18.7) | 13.0%  (12.0-14.1) | 1.20  (0.99-1.46) | 1.20  (0.98-1.46) |
| Togo | 13.0%  (9.8-17.0) | 7.6%  (6.5-8.9) | 1.73***  (1.28-2.32) | 1.71***  (1.27-2.30) | 8.1%  (5.3-12.2) | 4.5%  (3.6-5.6) | 1.83**  (1.17-2.86) | 1.83**  (1.20-2.79) |
| Afghanistan | 38.8%  (35.9-41.8) | 21.7%  (20.3-23.2) | 1.75***  (1.62-1.90) | 1.74***  (1.61-1.89) | 22.8%  (20.5-25.2) | 13.4%  (12.4-25.1) | 1.68***  (1.50-1.88) | 1.66***  (1.49-1.86) |
| Madagascar | 7.2%  (5.6-9.3) | 3.2%  (2.8-3.7) | 2.26***  (1.71-3.00) | 2.30***  (1.73-3.05) | 7.2%  (5.5-9.3) | 2.6%  (2.2-3.1) | 2.82***  (2.05-3.87) | 2.87***  (2.10-3.92) |
| DR Congo | 20.5%  (16.2-25.7) | 10.7%  (9.3-12.2) | 1.91***  (1.47-2.49) | 1.88***  (1.44-2.46) | 12.2%  (8.9-16.7) | 6.2%  (5.3-7.2) | 1.97***  (1.38-2.80) | 1.91***  (1.41-2.60) |
| Sierra Leone | 12.7%  (11.0-14.7) | 12.7%  (12.1-13.4) | 1.05  (0.89-1.23) | 1.04  (0.89-1.23) | 12.6%  (10.9-14.6) | 8.8%  (8.3-9.4) | 1.64***  (1.39-1.94) | 1.64***  (1.39-1.93) |
| Central African Republic | 23.6%  (20.1-27.5) | 13.4%  (11.9-15.1) | 1.75***  (1.44-2.12) | 1.75***  (1.44-2.12) | 15.7%  (12.5-19.4) | 8.0%  (6.8-9.3) | 1.95***  (1.50-2.53) | 1.94***  (1.49-2.53) |
| Malawi | 7.6%  (6.2-9.3) | 4.2%  (3.7-4.7) | 1.82***  (1.44-2.30) | 1.80***  (1.43-2.27) | 5.4%  (4.3-6.9) | 3.2%  (2.8-3.7) | 1.68***  (1.28-2.21) | 1.64***  (1.25-2.16) |
| Note: * p<0.05 ** p<0.01 *** p<0.001  APRR = adjusted prevalence rate ratio (model 1 adjusted for age and gender; model 1 adjusted for age, gender, relative household wealth and highest level of maternal education) | | | | | | | | |

| Supplementary Table 2: Prevalence of Comorbid Anxiety and Depression Among Young People with Disabilities | | | | |
| --- | --- | --- | --- | --- |
|  | Prevalence (with 95% CI) | | APRR (with 95% CI) | |
|  | Disability | No Disability | APRR1 | APRR2 |
| Argentina | 9.5% (4.2-20.0) | 1.6% (1.0-2.5) | 6.18*** (2.65-14.41) | 5.46*** (2.52-11.80) |
| Costa Rica | 3.8% (2.0-7.3) | 0.8% (0.4-1.6) | 4.50** (1.74-11.67) | 4.31** (1.55-11.98) |
| Montenegro | 10.4% (4.3-23.1) | 2.2% (1.4-3.5) | 6.43* (1.12-37.04) | 6.62* (1.42-30.93) |
| Dominican Republic | 5.0% (2.7-9.1) | 1.3% (1.0-1.6) | 3.90*** (2.00-7.60) | 3.80*** (1.93-7.46) |
| Cuba | 7.7% (2.4-22.2) | 3.9% (2.9-5.1) | 1.99 (0.65-6.09) | 1.80 (0.59-5.49) |
| Turkmenistan | 6.7% (3.1-14.0) | 0.1% (0.0-0.2) | 104.95*** (38.60-285.31) | 104.60*** (40.77-268.39) |
| Serbia | 3.5% (1.7-6.8) | 2.1% (1.6-2.9) | 1.68 (0.79-3.56) | 1.55 (0.71-3.45) |
| Guyana | 0.9% (0.3-3.0) | 0.3% (0.1-0.8) | 3.79 (0.84-17.15) | 5.00 (0.99-25.29) |
| Fiji | 3.8% (1.7-8.1) | 0.8% (0.4-1.6) | 5.15** (1.70-15.58) | 5.81** (2.16-15.62) |
| Belarus | 13.5% (7.5-22.9) | 1.2% (0.7-2.0) | 11.81*** (5.67-24.63) | 6.88*** (3.02-15.71) |
| North Macedonia | 2.8% (0.8-9.3) | 1.6% (0.6-3.8) | 2.44 (0.63-9.47) | 2.35 (0.53-10.58) |
| Tuvalu | 0.0% (0.0-7.1) | 0.4% (0.1-1.7) | n/a | n/a |
| Suriname | 3.6% (2.4-6.6) | 1.1% (0.6-1.9) | 5.45*** (3.16-9.40) | 5.60*** (3.18-9.87) |
| Iraq | 15.6% (12.2-19.8) | 4.0% (3.4-4.6) | 3.81*** (2.91-4.98) | 3.70*** (2.87-4.77) |
| Georgia | 9.5% (4.4-19.5) | 0.6% (0.3-1.0) | 16.21*** (6.81-38.60) | 16.52*** (7.05-38.78) |
| Kosovo | 13.0% (6.9-23.1) | 0.8% (0.4-1.5) | 16.59*** (6.21-44.30) | 14.78*** (6.43-34.02) |
| Tonga | 4.7% (1.4-14.5) | 1.7% (1.0-2.9) | 2.99 (0.77-11.69) | 2.98 (0.71-12.55) |
| Palestine | 6.4% (3.8-10.8) | 1.4% (1.1-1.9) | 4.24*** (2.29-7.84) | 4.19*** (2.24-7.82) |
| Samoa | 5.0% (2.5-10.0) | 12.0% (9.2-15.5) | 0.42* (0.19-0.91) | 2.41*** (1.54-3.78) |
| Algeria | 5.9% (4.4-7.9) | 3.5% (3.0-3.9) | 1.67** (1.26-2.30) | 1.67** (1.24-2.25) |
| Mongolia | 4.2% (1.8-9.4) | 0.4% (0.2-0.6) | 12.15*** (4.61-32.00) | 12.93*** (4.75-35.16) |
| Tunisia | 9.6% (7.2-12.4) | 2.7% (2.2-3.3) | 4.12*** (2.99-5.68) | 4.03*** (2.92-5.56) |
| Kiribati | 2.3% (1.2-4.6) | 1.2% (0.7-2.0) | 2.12 (0.88-5.09) | 2.06 (0.92-4.61) |
| Vietnam | 2.5% (0.6-9.5) | 0.2% (0.1-0.3) | 14.79*** (3.70-59.17) | 13.78*** (3.64-52.19) |
| Honduras | 3.2% (2.1-4.7) | 1.3% (1.0-1.7) | 2.45*** (1.56-3.84) | 2.41*** (1.54-3.78) |
| Ghana | 0.7% (0.4-1.2) | 2.6% (1.9-3.4) | 0.26*** (0.13-0.52) | 0.26*** (0.13-0.51) |
| Uzbekistan | 9.8% (5.7-16.3) | 1.7% (1.1-2.7) | 5.47*** (2.62-11.41) | 4.77*** (2.42-9.40) |
| Nigeria | 4.9% (3.4-6.9) | 4.6% (4.1-5.3) | 1.04 (0.71-1.52) | 1.00 (0.67-1.47) |
| Sao Tome & Principe | 4.0% (2.1-7.7) | 1.8% (1.2-2.6) | 2.21 (0.98-4.96) | 2.12 (0.93-4.83) |
| Zimbabwe | 3.5% (1.6-7.2) | 0.8% (0.6-1.2) | 4.32** (1.86-10.04) | 4.24** (1.72-10.46) |
| Bangladesh | 8.2% (6.8-9.9) | 2.2% (2.0-2.5) | 3.69*** (2.99-4.56) | 3.80*** (3.07-4.70) |
| Lesotho | 1.4% (0.4-4.8) | 0.4% (0.2-0.6) | 4.14* (1.10-15.53) | 4.70* (1.29-17.11) |
| Kyrgyz Republic | 4.9% (1.5-10.4) | 0.5% (0.2-0.9) | 5.50*** (2.43-12.45) | 4.60** (1.92-11.01) |
| Nepal | 3.3% (1.9-5.8) | 1.9% (1.5-2.4) | 1.75 (0.96-3.19) | 1.71 (0.92-3.15) |
| Guinea-Bissau | 3.3% (1.0-10.0) | 4.2% (3.4-5.3) | 0.76 (0.23-2.52) | 0.64 (0.19-2.09) |
| The Gambia | 0.5% (0.2-1.5) | 0.8% (0.5-1.3) | 0.56 (0.17-1.86) | 0.57 (0.17-1.90) |
| Chad | 10.2% (8.0-13.0) | 10.3% (9.4-11.4) | 0.99 (0.77-1.27) | 0.99 (0.77-1.27) |
| Togo | 5.7% (3.5-9.1) | 3.7% (2.9-4.7) | 1.56 (0.99-2.44) | 1.55 (1.00-2.42) |
| Afghanistan | 19.2% (17.1-21.5) | 11.1% (10.1-12.2) | 1.70*** (1.50-1.93) | 1.68*** (1.49-1.91) |
| Madagascar | 3.0% (1.9-4.7) | 1.5% (1.2-1.8) | 2.12** (1.29-3.49) | 2.14** (1.30-3.54) |
| DR Congo | 10.9% (7.6-15.5) | 4.9% (4.1-5.9) | 2.24*** (1.61-3.12) | 2.19*** (1.58-3.05) |
| Sierra Leone | 8.9% (7.2-10.9) | 6.2% (5.7-6.8) | 1.65*** (1.36-2.00) | 1.65*** (1.36-2.00) |
| Central African Republic | 10.7% (8.3-13.7) | 6.0% (4.9-7.3) | 1.78*** (1.32-2.41) | 1.79*** (1.31-2.43) |
| Malawi | 4.2% (3.2-5.5) | 2.5% (2.2-3.0) | 1.64*** (1.19-2.26) | 1.62** (1.18-2.23) |
